# Association of Tumor-Infiltrating Lymphocytes With Survival in Stages II and III Colorectal Cancer

**DOI:** 10.7759/cureus.31144

**Published:** 2022-11-06

**Authors:** Marina Vitorino, Inês Eiriz, Tiago C Tomás, Rodrigo Vicente, Ana Mendes, Ana Rita Freitas, Catarina Alves-Vale, André Ferreira, Luisa Leal da Costa, Sofia Braga, Paula Borralho

**Affiliations:** 1 Oncology, Hospital Professor Doutor Fernando Fonseca, Lisbon, PRT; 2 Pathology, CUF Oncologia, Lisbon, PRT; 3 Medical Oncology, Hospital de São Francisco Xavier, Lisbon, PRT; 4 Oncology, Hospital Beatriz Ângelo, Loures, PRT

**Keywords:** prognosis, tumour microenvironment, survival, immunity, tumour-infiltrating lymphocytes, colorectal cancer

## Abstract

The tumor microenvironment is crucial in tumourigenesis, response to therapy, and elimination of tumor cells. Tumor-infiltrating lymphocytes (TILs) promote the host immune response and are associated with a better prognosis in colorectal cancer (CRC). This multicentric retrospective study evaluated the relationship between the presence and intensity of TILs and survival outcomes. A total of 651 patients from four Portuguese oncological centers who underwent surgical resection for stages II or III colorectal adenocarcinoma between 2016 and 2019 were included in this study. The mean age of the study population was 70 years; 58.2% were males. The median overall survival was 58.03 ± 1.29 months (95% confidence interval (CI) 55.50 - 60.56), and the median disease-free survival (DFS) was 53.02 ± 1.39 months (95% CI 50.29 - 55.74). Patients with high infiltrate (including those with moderate, abundant, or Crohn-like infiltrate) had significantly longer DFS i.e., 58.48 ± 1.84 months (95% CI 54.87 - 62.09 months) vs 49.22 ± 1.75 months (95% CI 45.79 - 52.64 months) in the group with absent or minimal infiltrate; p = 0.003. Assessing the side of the tumor, high infiltrate was associated with higher DFS (59.86 ± 2.36 months (95% CI 55.23 - 64.50 months) vs 49.60 ± 2.40 months (95% CI 44.90 - 54.29 months), p = 0.011). This work reinforces the importance of research into possible prognostic and predictive factors in patients with CRC.

## Introduction

Colorectal cancer (CRC) is the fourth most common malignant disease and the third leading cause of cancer deaths worldwide. In 2020, there were an estimated 1.9 million newly diagnosed CRC patients and 935,000 CRC-related deaths worldwide [1].

There are well-known clinical and histological prognostic factors for CRC. However, it is increasingly clear that the tumor microenvironment (TME) plays a role in the prognosis and response to therapy in solid tumors [2]. The TME has the capacity to interact with tumor cells, eliminating them or making them more susceptible to the action of anti-neoplastic agents. Recent studies have demonstrated that the density of immune cell infiltration in the TME of CRC is related to disease prognosis [3]. The presence of a higher density of tumor-infiltrating lymphocytes (TILs) is a marker of immune activation of the environment surrounding the tumor and is associated with a better prognosis in CRC. Despite the demonstrated association of the presence of TILs with outcomes in CRC, more recent studies have focused the prognostic analysis mainly on the presence of microsatellite instability (MSI) [4]. The presence of TILs is more common in tumors with high levels of MSI (MSI-H) than in microsatellite-stable (MSS) tumors [5]. Tumors with MSI-H accumulate inserts and deletions in DNA repeat sequences with a high mutational load leading to the production of many neoantigens recognized by the immune system, which can trigger the lymphocytic infiltrates [3]. Concerning the assessment of TILs, in previous studies, different TIL assessment scales have been used, making it difficult to conduct a uniform assessment.

In this study, the authors retrospectively evaluated data on CRC patients to assess the relationship between the presence and intensity of TILs and survival outcomes.

## Materials and methods

This multicentric retrospective study included 651 patients from four Portuguese oncological centers (Hospital Professor Doutor Fernando Fonseca, Centro Hospitalar Lisboa Ocidental, Hospital Beatriz Ângelo, and Lisbon CUF Hospitals). All patients were >18 years of age and underwent surgical resection for stage II or III colorectal adenocarcinoma during the calendar years 2016 to 2019.

Exclusion criteria included patients who underwent biopsy or endoluminal treatment only and an absence of detailed information in histopathologic reports. When multiple tumors were present, the highest-stage tumor was assessed. Further data on histological diagnosis, histological grade, pathological tumor-node-metastasis (TNM) stage, and tumor location (evaluated according to the WHO Classification of Tumors, 5th Edition, and the 8th Edition American Joint Committee on Cancer Staging Manual, respectively) were obtained from the reports. The TIL grade score (routinely classified as absent, minimal, moderate, and abundant, following the recommendations from the International TILs Working Group) was also retrieved from the pathological report [4,6]. When prominent and with lymphoid aggregates, the infiltrate was considered Crohn-like [7]. The peri and intratumoral inflammatory infiltrate were considered in two categories for statistical analysis: 1) the high infiltrate group included cases with moderate, abundant, or Crohn-like infiltrate, and 2) the absent/minimal infiltrate group included absent and minimal infiltrate. Demographic, clinical, surgical, and additional histopathological variables were also retrieved.

Statistical analysis involved descriptive statistics (absolute and relative frequencies) and inferential statistics. Fisher’s test, logistic regression, Cox regression, and Kaplan-Meier survival analysis were used. The level of significance to reject the null hypothesis was set at (α) ≤ 0.05. Statistical analysis was performed with the Statistical Package for the Social Sciences (SPSS) version 28.0 (IBM Corp., Armonk, NY, USA). Overall survival (OS) was defined as the time from diagnosis to death due to any cause, and disease-free survival (DFS) was defined as the time from diagnosis to the first of either disease recurrence or death (date of the census: December 1, 2021). The survival analyses were performed using the presence of high or low/absent TILs scores. This study was approved by the Ethical Committee for Health of Hospital Professor Doutor Fernando Fonseca (approval No. 099/2020).

## Results

The demographic characteristics of the study population are described in Table 1. A total of 651 patients were included; the mean age was 70 years (range: 25 to 96 years), and 58.2% were males (n = 379). All tumors corresponded to adenocarcinoma. Three hundred forty patients (52.3%) had left-sided colon tumors, 218 (33.5%) had right-sided colon tumors, and 92 (14.2%) had rectal tumors. Three hundred seventeen patients (48.9%) were classified as stage II, and 331 (51.1%) as stage III. One hundred seventy-seven (27.2%) patients had high-grade tumors, 270 (41.5%) had vascular invasion, and 145 (22.3%) had a perineural invasion. Tumour obstruction or perforation occurred in 157 (24.1%) patients. Among the 651 patients, 333 (52.3%) were treated with adjuvant chemotherapy.

**Table 1:** Demographic characteristics of the study population

Characteristics	N (total=651)
Age at diagnosis in years, mean (minimum to maximum)	70 (25–96)
Male, n (%)	379 (58.2%)
Location of tumor	
Right colon	218 (33.5%)
Left colon	340 (52.3%)
Rectal	92 (14.2%)
Staging	
Stage II	317 (48.9%)
Stage III	331 (51.1%)
Histologic grade*	
Low	469 (72.0%)
High	177 (27.2%)
Vascular invasion	270 (41.5%)
Perineural invasion	145 (22.3%)
Tumour perforation or obstruction	157 (24.1%)
Adjuvant treatment	333 (52.3%)
Stage II	93 (27.9%)
Stage III	240 (72.1%)
* 5 missing values	

Not all pathology reports described the inflammatory infiltrate with the terms “peritumoral infiltrate” and “intratumoral infiltrate”. A total of 16 missing values for "peritumoral infiltrate" and 229 for "intratumoral infiltrate" were detected. A total of 259 patients (39.8%) were classified as high infiltrate (moderate, abundant, or Crohn-like infiltrate).

Survival analyses

The median OS was 58.03 ± 1.29 months (95% confidence interval (CI) 55.50 - 60.56) and the median disease-free survival (DFS) was 53.02 ± 1.39 months (95% CI 50.29 - 55.74).

**Figure 1:**
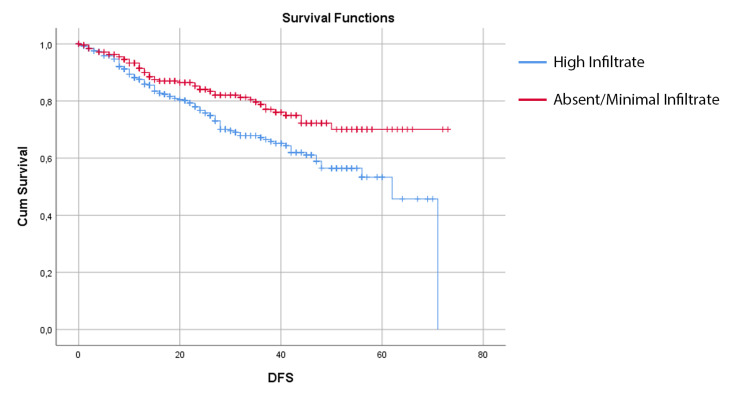
Kaplan-Meier estimates of disease-free survival for colorectal cancer patients with high infiltrate and absent/minimal infiltrate

The presence of high infiltrate was associated with better survival outcomes. There was a significant association between the presence of high infiltrate and DFS. The mean DFS in the high infiltrate group was 58.48 ± 1.84 (95% CI 54.87 - 62.09), compared with 49.22 ± 1.75 (95% CI 45.79 - 52.64) in the group with absent/minimal infiltrate (p = 0.003) (Figure 1).

Although the mean OS in the high infiltrate group was superior compared with the absent/minimal infiltrate group (59.88 vs 56.85 months), a statistically significant difference was not found (p = 0.432).

**Figure 2:**
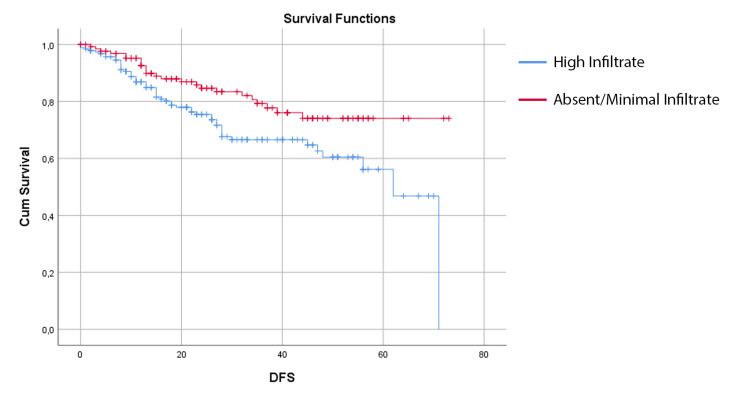
Kaplan-Meier estimates of disease-free survival for left colon cancer patients with high infiltrate and absent/minimal infiltrate

**Figure 3:**
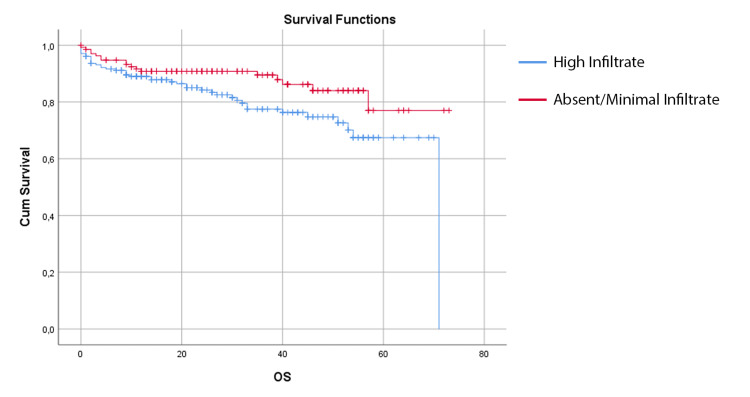
Kaplan-Meier estimates of overall survival for left colon cancer patients with high infiltrate and absent/minimal infiltrate

Assessing the right and left colon cancer patients, we found that the presence of high infiltrate was associated with better survival outcomes in left, but not in right colon cancer. In this group of patients, the mean DFS in the high infiltrate group was 59.86 ± 2.36 months (95% CI 55.23 - 64.50 months), compared with 49.60 ± 2.40 months (95% CI 44.90 - 54.29 months) in the group with absent or minimal inflammatory infiltrate (p = 0.011) (Figure 2). Also, the OS is better in the high infiltrate group for the left colon, with a mean OS 63.50 ± 2.19 months (95% CI 59.22 - 67.79 months) compared with 56.24 ± 2.03 months (95% CI 52.26 - 60.23 months) in the group without inflammatory infiltrate (p = 0.028) (Figure 3).

Assessing the staging of the tumor, we found different results in stage II compared with stage III patients. In stage II, high infiltrate was associated with a better DFS (57.53 ± 1.73 (95% CI 54.15 - 60.91) compared with the absent or minimal inflammatory infiltrate (51.02 ± 1.90 (95% CI 47.30 - 54.75); p = 0.037). In stage III patients, although the DFS in the high infiltrate group was superior compared with the absent/minimal infiltrate group (52.16 vs 45.01 months), a statistically significant difference was not found (p = 0.106). Regarding OS evaluation, no significant differences were found in either stage. In addition, in univariate analysis, the staging and presence of perineural invasion were significantly associated with DFS, but not with OS.

## Discussion

Most CRC diagnoses occur at early stages, allowing for better survival. However, after resection with curative intent, approximately 20% of patients with stage II or III CRC will have a disease recurrence. Certain well-documented clinical and histological risk factors are associated with a higher probability of recurrence. In stage III patients and those in stage II with risk factors, adjuvant treatment is recommended to reduce the recurrence risk [8].

Our study describes the clinical and histological characteristics of stage II and III CRC patients who underwent surgical resection in four Portuguese institutions over four years. As expected, initial tumor staging was identified as a prognostic factor in our sample, with stage III being associated with worse DFS compared to stage II. The OS was not significantly different among stage III patients, likely due to the short follow-up time.

Since the 1970s, evaluating the immune environment around the tumor has been described as an essential factor in CRC prognosis. The immune response is an important factor in tumor behavior and affects tumor development and growth. Infiltration of immune cells such as lymphocytes, macrophages, and dendritic cells predicts the immune response, promoting tumor elimination [3,9]. The location, density, and types of cells in the TME are relevant to the prognosis of CRC patients. The inflammatory infiltrates may be in the tumor itself or the surrounding stroma. The peritumoral infiltrate is described according to a semiquantitative method on hematoxylin and eosin (H&E) slides. The most commonly used scoring systems are the Klintrup-Makinen grade and the Jass score [10]. Watt et al. reported that perivascular and peritumoral lymphoid infiltration was related to higher survival rates [11,12]. However, these elements are not currently mentioned in the protocol for assessing primary CRC specimens from the College of American Pathologists, despite being suggested by the Royal College of Pathologists as a non-core data item [13,14].

The lymphocyte subpopulations in the TME in CRC allowed the establishment of response patterns. A multiplex immunohistochemical method identifies specific immune cells according to the surface markers. Cells such as regulatory T cells and M2 tumor-associated macrophages play mostly tumor-promoting functions. On the other hand, macrophages of M1 phenotypes or T cells CD8+ are related to antitumor activity, driving a better prognosis [15]. Several studies have analyzed and quantified lymphocyte infiltration in CRC patients, associating it with better survival outcomes [16]. However, different methodologies, quantification methods, definitions of outcomes, and samples were used in these studies [4,17,18]. A meta-analysis by Idos et al. brought together various studies to clarify the role of TILs in CRC [19].

In our study, the lymphocytic infiltrates were evaluated according to anatomopathological reports previously made. This limits the uniformity of the evaluation as it is subject to inter-observer variation. Despite these limitations, the benefit of the presence of inflammatory infiltrates in TME is notable, highlighting the importance of routinely reporting this variable for colon cancer surgical specimens in clinical practice. The increase in DFS was almost 10 months in the patients with high infiltrate compared to those with absent/minimal infiltrate.

In most recent studies, the presence of MSI has been reported as a prognostic factor in CRC patients. In addition, MSI-H is associated with a higher response rate with immune checkpoint inhibitors [3,16,20]. The density of TILs is frequently high in MSI-H tumors, in right-sided colon tumors, and in tumors that harbor a BRAF mutation. The presence of more abundant TILs in MSI-H tumors can be explained by the production of neoantigens consequent to an impaired DNA damage repair system [2,21]. The absence of data on MSI and BRAF status is a limitation of this study; this information was not available in the pathological reports as these measures were not assessed routinely during the study period.

The tumor side is a known prognostic factor given the differences in the biological origin of the tumors. The tumor location also has therapeutic implications, mostly related to anti-epidermal growth factor receptor antibodies [22]. In our sample, no differences in survival outcomes were associated with the location of the tumor. However, in the left-sided colon cancer group, we found a significant difference in survival outcomes between the group with high infiltrate and the group with absent/minimal infiltrate (as seen above in Figures 2, 3).

Our study confirms the prognostic importance of inflammatory infiltrates in CRC tumors, with significant differences observed in DFS. The study's limitations include the fact that it is a retrospective analysis that depends on previous pathological reports. In future studies, it would be valuable to include pathological re-analysis (e.g., the verification of MSI and BRAF mutational status) as well as an increased sample size involving the participation of other centers.

## Conclusions

This work reinforces the importance of research into possible prognostic and predictive factors in patients with CRC. Identifying factors that alter prognosis has implications for the type of surveillance to be performed and for the possibility of adjuvant treatment. The study of the TME is a critical factor in understanding oncological treatment. The development of methods that evaluate components of the immune system in the tumor stroma allows the relationship between their presence and responses to therapies and survival outcomes to be assessed.

In our study, the presence of TILs was significantly associated with DFS. The results obtained are concordant with what has been previously reported in individual studies and meta-analyses. However, there is a need for further research on this topic, including new markers that are easily accessible in clinical practice.

## References

[1] Sung H, Ferlay J, Siegel RL, Laversanne M, Soerjomataram I, Jemal A, Bray F (2021). Global Cancer Statistics 2020: GLOBOCAN estimates of incidence and mortality worldwide for 36 cancers in 185 countries. CA Cancer J Clin.

[2] Zhao Y, Ge X, He J, Cheng Y, Wang Z, Wang J, Sun L (2019). The prognostic value of tumor-infiltrating lymphocytes in colorectal cancer differs by anatomical subsite: a systematic review and meta-analysis. World J Surg Oncol.

[3] Bai Z, Zhou Y, Ye Z, Xiong J, Lan H, Wang F (2021). Tumor-infiltrating lymphocytes in colorectal cancer: the fundamental indication and application on immunotherapy. Front Immunol.

[4] Fuchs TL, Sioson L, Sheen A (2020). Assessment of tumor-infiltrating lymphocytes using international TILs working group (ITWG) system is a strong predictor of overall survival in colorectal carcinoma: a study of 1034 patients. Am J Surg Pathol.

[5] Ferris RL, Galon J (2016). Additional support for the introduction of immune cell quantification in colorectal cancer classification. J Natl Cancer Inst.

[6] Salgado R, Denkert C, Demaria S (2015). The evaluation of tumor-infiltrating lymphocytes (TILs) in breast cancer: recommendations by an International TILs Working Group 2014. Ann Oncol.

[7] Rozek LS, Schmit SL, Greenson JK, Tomsho LP, Rennert HS, Rennert G, Gruber SB (2016). Tumor-infiltrating lymphocytes, Crohn's-like lymphoid reaction, and survival from colorectal cancer. J Natl Cancer Inst.

[8] Bae JM, Yoo SY, Kim JH, Kang GH (2020). Immune landscape and biomarkers for immuno-oncology in colorectal cancers. J Pathol Transl Med.

[9] Galon J, Costes A, Sanchez-Cabo F (2006). Type, density, and location of immune cells within human colorectal tumors predict clinical outcome. Science.

[10] Wilkinson K, Ng W, Roberts TL, Becker TM, Lim SH, Chua W, Lee CS (2021). Tumour immune microenvironment biomarkers predicting cytotoxic chemotherapy efficacy in colorectal cancer. J Clin Pathol.

[11] Watt AG, House AK (1978). Colonic carcinoma. A quantitative assessment of lymphocyte infiltration at the periphery of colonic tumors related to prognosis. Cancer.

[12] Pihl E, Malahy MA, Khankhanian N, Hersh EM, Mavligit GM (1977). Immunomorphological features of prognostic significance in Dukes' Class B colorectal carcinoma. Cancer Res.

[13] Loughrey MB, Quirke P, Shepherd NA (2019). Dataset for histopathological reporting of colorectal cancer. http://2018.

[14] Washington MK, Berlin J, Branton P (2009). Protocol for the examination of specimens from patients with primary carcinoma of the colon and rectum. Arch Pathol Lab Med.

[15] Czajka-Francuz P, Cisoń-Jurek S, Czajka A, Kozaczka M, Wojnar J, Chudek J, Francuz T (2021). Systemic interleukins' profile in early and advanced colorectal cancer. Int J Mol Sci.

[16] Alsalman A, Al-Mterin MA, Murshed K, Alloush F, Al-Shouli ST, Toor SM, Elkord E (2022). Circulating and tumor-infiltrating immune checkpoint-expressing CD8+ Treg/T cell subsets and their associations with disease-free survival in colorectal cancer patients. Cancers (Basel).

[17] Hendry S, Salgado R, Gevaert T (2017). Assessing tumor-infiltrating lymphocytes in solid tumors: a practical review for pathologists and proposal for a standardized method from the international immuno-oncology biomarkers working group: part 2: TILs in melanoma, gastrointestinal tract carcinomas, non-small cell lung carcinoma and mesothelioma, endometrial and ovarian carcinomas, squamous cell carcinoma of the head and neck, genitourinary carcinomas, and primary brain tumors. Adv Anat Pathol.

[18] Huh JW, Lee JH, Kim HR (2012). Prognostic significance of tumor-infiltrating lymphocytes for patients with colorectal cancer. Arch Surg.

[19] Idos GE, Kwok J, Bonthala N, Kysh L, Gruber SB, Qu C (2020). The prognostic implications of tumor-infiltrating lymphocytes in colorectal cancer: a systematic review and meta-analysis. Sci Rep.

[20] Liao X, Li G, Cai R, Chen R (2022). A review of emerging biomarkers for immune checkpoint inhibitors in tumors of the gastrointestinal tract. Med Sci Monit.

[21] Kim JK, Chen CT, Keshinro A (2022). Intratumoral T-cell repertoires in DNA mismatch repair-proficient and -deficient colon tumors containing high or low numbers of tumor-infiltrating lymphocytes. Oncoimmunology.

[22] Saberzadeh-Ardestani B, Foster NR, Lee HE, Shi Q, Alberts SR, Smyrk TC, Sinicrope FA (2022). Association of tumor-infiltrating lymphocytes with survival depends on primary tumor sidedness in stage III colon cancers (NCCTG N0147) [Alliance]. Ann Oncol.

