# Correction: Association of Tumor-Infiltrating Lymphocytes With Survival in Stages II and III Colorectal Cancer

**DOI:** 10.7759/cureus.c91

**Published:** 2023-01-09

**Authors:** Marina Vitorino, Inês Eiriz, Tiago C Tomás, Rodrigo Vicente, Ana Mendes, Ana Rita Freitas, Catarina Alves-Vale, André Ferreira, Luisa Leal da Costa, Sofia Braga, Paula Borralho

**Affiliations:** 1 Oncology, Hospital Professor Doutor Fernando Fonseca, Lisbon, PRT; 2 Pathology, CUF Oncologia, Lisbon, PRT; 3 Medical Oncology, Hospital de São Francisco Xavier, Lisbon, PRT; 4 Oncology, Hospital Beatriz Ângelo, Loures, PRT

This article has been corrected at the request of the authors due to an error in the author order when originally published. The authors deeply regret that this error was not identified and corrected prior to publication. The correct author order is now shown.

